# Ecological disturbance reduces genomic diversity across an Alpine whitefish adaptive radiation

**DOI:** 10.1111/eva.13617

**Published:** 2023-11-23

**Authors:** David Frei, Salome Mwaiko, Ole Seehausen, Philine G. D. Feulner

**Affiliations:** ^1^ Department of Fish Ecology and Evolution EAWAG Swiss Federal Institute of Aquatic Science and Technology Kastanienbaum Switzerland; ^2^ Division of Aquatic Ecology and Evolution, Institute of Ecology and Evolution University of Bern Bern Switzerland

**Keywords:** Alpine whitefish, anthropogenic environmental change, genomic diversity decline

## Abstract

Genomic diversity is associated with the adaptive potential of a population and thereby impacts the extinction risk of a species during environmental change. However, empirical data on genomic diversity of populations before environmental perturbations are rare and hence our understanding of the impact of perturbation on diversity is often limited. We here assess genomic diversity utilising whole‐genome resequencing data from all four species of the Lake Constance Alpine whitefish radiation. Our data covers a period of strong but transient anthropogenic environmental change and permits us to track changes in genomic diversity in all species over time. Genomic diversity became strongly reduced during the period of anthropogenic disturbance and has not recovered yet. The decrease in genomic diversity varies between 18% and 30%, depending on the species. Interspecific allele frequency differences of SNPs located in potentially ecologically relevant genes were homogenized over time. This suggests that in addition to the reduction of genome‐wide genetic variation, the differentiation that evolved in the process of adaptation to alternative ecologies between species might have been lost during the ecological disturbance. The erosion of substantial amounts of genomic variation within just a few generations in combination with the loss of potentially adaptive genomic differentiation, both of which had evolved over thousands of years, demonstrates the sensitivity of biodiversity in evolutionary young adaptive radiations towards environmental disturbance. Natural history collections, such as the one used for this study, are instrumental in the assessment of genomic consequences of anthropogenic environmental change. Historical samples enable us to document biodiversity loss against the shifting baseline syndrome and advance our understanding of the need for efficient biodiversity conservation on a global scale.

## 1 INTRODUCTION

Genetic diversity represents the most fundamental level of biodiversity. Genomic diversity is central to sustain viable populations and to preserve evolutionary potential, enabling the adaptation to changing environmental conditions (Hoffmann et al., 2017; Willi et al., 2022). As a consequence, genomic diversity is one key component determining the extinction risk of a population during environmental change (Jensen & Leigh, 2022). Disturbance of ecosystems can influence not only genomic variation through both selective but also demographic (and selectively neutral) processes, as well as the interaction of both (Banks et al., 2013). As a result, the history of environmental disturbance may be a major driver shaping patterns and dynamics of genomic diversity in many natural systems (Banks et al., 2013). As both the frequency and strength of anthropogenic ecological disturbances are increasing (IPBES, 2019; Turner, 2010), it is essential to advance our understanding of how such disturbance affects biodiversity at its most basal level, which is genetic and/or genomic diversity (Banks et al., 2013).

Anthropogenic eutrophication during the last century had dramatic consequences on the biodiversity of many perialpine lakes in Switzerland (Feulner & Seehausen, 2019; Frei, De‐Kayne, et al., 2022; Vonlanthen et al., 2012). The effects on many species of the Alpine whitefish were particularly detrimental. In total, about a third of the more than 30 taxonomically described whitefish species went extinct during the period of anthropogenic eutrophication (Selz et al., 2020; Steinmann, 1950; Vonlanthen et al., 2012). High‐nutrient inputs altered many habitat characteristics of the deep and oligotrophic Swiss lakes, affecting both diet and reproduction of many whitefish species (Vonlanthen et al., 2012). The loss of suitable, well‐oxygenated spawning grounds together with the shift in food resources resulted in the extinction of multiple species through a combination of demographic decline and speciation reversal through introgressive hybridization (Frei, De‐Kayne, et al., 2022; Vonlanthen et al., 2012). The improvement of sewage treatment and phosphorus management towards the end of the last century resulted in many of the Swiss lakes returning close to their natural oligotrophic state (Vonlanthen et al., 2012). Even though the changed environmental conditions were transient and of relatively short duration, the period of cultural eutrophication had severe consequences on the genomic variation of the Alpine whitefish radiation (Frei, De‐Kayne, et al., 2022).

In evolutionary young adaptive radiations, such as the Alpine whitefish radiation, sympatric species are still able to hybridize (Schluter, 2000; Seehausen et al., 2008). This is because complete reproductive isolation takes orders of magnitudes longer to evolve than the rapid speciation events in such young radiations (Schluter, 2009; Seehausen et al., 2008). The ability to exchange genomic variation might become particularly important during ecological disturbance: When environmental conditions rapidly change into an unfavorable state for a certain species of a young adaptive radiation, habitats can be lost and food resources might become unavailable, resulting in demographic decline. The decreasing population size is strengthening genetic drift, reducing genetic diversity in the declining population. In such a situation, the exchange of genomic variation with other members of the adaptive radiation through hybridization could become beneficial (Frei, Reichlin, et al., 2022; Grant & Grant, 2019). Hybridization might increase genomic variation of the population and enhance its evolvability, increasing the likelihood of adaptation to the changed environmental conditions through evolutionary rescue (Gilman & Behm, 2011). In order to document the effects of ecological disturbance on genomic variation following natural disturbance, genomic time‐series data capturing the disturbance event are essential (Jensen & Leigh, 2022).

The Lake Constance whitefish radiation was strongly affected by anthropogenic eutrophication during the last century. Using historical fish scale samples, previous work demonstrated that all four taxonomically described whitefish species of Lake Constance (*Coregonus gutturosus*, *C. arenicolus*, *C. macrophthalmus*, *C. wartmanni*) extensively hybridized during the eutrophication period (Frei, De‐Kayne, et al., 2022; Vonlanthen et al., 2012). One species (*C. gutturosus*) went extinct by a combination of demographic decline and speciation reversal through introgressive hybridization during the period of anthropogenic eutrophication (Frei, De‐Kayne, et al., 2022; Vonlanthen et al., 2012), and according to fisheries management, population sizes of all Lake Constance whitefish dramatically decreased over the last decades (Alexander & Seehausen, 2021). The potential to generate temporal whole‐genome resequencing data spanning the entire eutrophication event and including four species makes the Lake Constance whitefish radiation an outstanding system to study the effects of ecological disturbance on genomic diversity.

Previous work focused on speciation reversal and the genomic consequences of hybridization between all species within the Lake Constance Alpine whitefish radiation (Frei, De‐Kayne, et al., 2022). Here we evaluate changes of genomic diversity within species over time. We assess if and to which extent intraspecies genomic diversity declined besides hybridization between species. Furthermore, while previous work highlighted the potential of adaptative introgression of alleles from an extinct species into contemporary species (Frei, De‐Kayne, et al., 2022; Frei, Reichlin, et al., 2022), we here explore genomic patterns consistence with a loss of potentially functionally relevant variation over time. To do so, we used natural history collections to sequence population scale data (5–12 whole genomes per population) of each of the four Lake Constance whitefish species before the onset of the anthropogenic eutrophication (before 1950), as well as data from all three extant species during the peak eutrophication period (1970–1980). In combination with existing sequencing data from the three surviving species (*N* = 6–13) collected after the eutrophication period ended and the lake returned to an oligotrophic state, we produced a time‐series data set capturing the whole period (pre, during and post) of anthropogenic eutrophication. During this period of anthropogenic ecological disturbance, we observed a strong decline in genomic diversity over time and found genomic signals of population declines in all species. By establishing a baseline of genomic diversity before the occurrence of an anthropogenic disturbance, our work also demonstrated the value of natural history collection for biodiversity research.

## 2 METHODS

### 2.1 Sample collection

Historical whitefish scale samples previously used in Vonlanthen et al. (2012) and Frei, De‐Kayne, et al. (2022) were used to extract DNA from 12 individuals of each population (pre‐ and during‐eutrophication) of each species (see Table S1). These samples were collected from fisheries authorities around the lake during the last century and have been assembled by David Bittner (see Vonlanthen et al., 2012 for details). For the post‐eutrophication populations, we used sequencing data (sampled 2015) produced by Frei, De‐Kayne, et al. (2022) retrieved from ENA with accession PRJEB43605, as well as data from Frei, Reichlin, et al. (2022) (sampled 2019) retrieved from ENA with accession PRJEB53050 (see Table S1 for the 112 sample accessions).

### 2.2 DNA extraction and sequencing

DNA was extracted according to Frei, De‐Kayne, et al. (2022). In brief, DNA extraction of historical scale samples was done using the Qiagen DNeasy blood and tissue kit (Qiagen AG, CH). For scale samples, we followed the manufacturer's protocol for crude lysates with minor adjustments (alternative lysis buffer from Wasko et al., 2003 containing 4M UREA and overnight incubation at 37°C).

Libraries were produced using the Accel‐NGS 1S Plus DNA library kit (Swift Biosciences) at the NGS platform of the University of Bern. Libraries were then sequenced paired‐end 100 bp on a Novaseq 6000 S4 flowcell.

### 2.3 Data processing

We removed poly‐G strings with fastp (Chen et al., 2018) and then merged overlapping read pairs (with overlaps longer than 25 bp) using SeqPrep 1.0. (https://github.com/jstjohn/SeqPrep). The processed reads were then aligned to the Alpine whitefish reference genome (De‐Kayne et al., 2020) with bwa mem version 0.7.12 (Li & Durbin, 2009) and adjusting the “r” parameter to 1. Finally, we used picard‐tools (Version 2.20.2; http://broadinstitute.github.io/picard/) to mark duplicate reads (MarkDuplicates), fix mate information (FixMateInformation) and replace read groups (AddOrReplaceReadGroups).

### 2.4 Population genomic analysis

Genotype likelihoods at polymorphic sites were calculated using angsd 0.925 (Korneliussen et al., 2014), using the samtools genotype likelihood model. For that purpose, we excluded reads with a mapping quality below 30, bases with base qualities below 20 and reads that did not map uniquely to the reference. Only sites passing a *p*‐value cut‐off of 10E‐6 for being variable, with a sequencing depth above 2× in each individual and with data of at least 80 of all 112 individuals were included. Additionally, we only analysed whitefish chromosomes without any potentially collapsed duplicated regions (De‐Kayne et al., 2020) to avoid potential bias due to imbalanced ploidy levels (also note that there is no evidence for a heterogametic sex chromosome in Alpine whitefish). We applied SNP filters to avoid strand bias (‐sb_pval 0.05), quality score bias (‐qscore_pval 0.05), edge bias (‐edge_pval 0.05) and mapping quality bias (‐mapq_pval 0.05). This resulted in a total of 355,311 polymorphic sites for further analysis.

To verify the species assignment done in the field when these samples have been collected by fisheries authorities, we performed a PCA using PCAngsd 1.02 (Meisner & Albrechtsen, 2018). We excluded sites with a minor allele frequency below 0.05 (across the whole dataset), resulting in 128,164 sites. Default parameters were used, except for using the first three eigenvectors to estimate individual allele frequencies (‐e 3). By this PCA approach, we identified 12 individuals suggesting an erroneous species assignment (in the field) which were excluded from all subsequent analyses (one post‐eutrophication *C. wartmanni* was genetically assigned to *C. arenicolus*, and another one to *C. macrophthalmus*; one post‐eutrophication and six during‐eutrophication *C. macrophthalmus* samples were genetically assigned to *C. wartmanni*, one during‐eutrophication *C. macrophthalmus* was assigned to *C. arenicolus* and two pre‐eutrophication *C. macrophthalmus* were assigned to *C. gutturosus*; see Figure S1 and Table S1).

Based on the genotype likelihoods inferred in the remaining 100 samples (excluding 12 individuals potentially misidentified in the field), we calculated Watterson's theta (*θ*_ω_) and Tajima's *D* in 100 kb windows along the genome (Korneliussen et al., 2013). To do this, the folded site allele frequency likelihood for each species and each sampling timepoint separately (*N* = 5–13; see Table S2) was calculated in angsd (0.925) (Korneliussen et al., 2014; Nielsen et al., 2012). The maximum likelihood estimate of the folded site allele frequency spectrum was inferred using realSFS of angsd (0.925) (Korneliussen et al., 2014). With the global site allele frequency spectrum, we calculated different theta estimators and Tajima's *D* in 100 kb windows using thetaStat of angsd (0.925) (Korneliussen et al., 2013, 2014). We used all 100 kb windows to calculate a genome wide average.

We used NgsRelate v2 (Hanghøj et al., 2019; Korneliussen & Moltke, 2015) to calculate pairwise relatedness between all individuals of each species at each sampled timepoint based on genotype likelihoods. We split the genotype likelihood file generated across all species and timepoints into each single species and timepoints, and used these separate genotype likelihood fields as input to NgsRelate v2 (Hanghøj et al., 2019; Korneliussen & Moltke, 2015). At each polymorphic site in the genotype likelihood file, we calculated the allele frequency in each species at each timepoint in angsd 0.925 (Korneliussen et al., 2014) using the method from Kim et al. (2011), and also used this allele frequency information as input to NgsRelate v2 (Hanghøj et al., 2019; Korneliussen & Moltke, 2015), which we then used to calculate pairwise relatedness with default parameters. We finally calculated the mean relatedness in each species and timepoint by averaging across all pairwise relatedness values for each population in R (R Core Team, 2018).

For each of the 355,311 polymorphic sites, we calculated the weighted *F*_ST_ between each species and all other species pooled together (of only the pre‐eutrophication populations) in angsd 0.925 (Korneliussen et al., 2014) from one‐ and two‐dimensional site frequency spectra which were inferred from site allele frequencies (Nielsen et al., 2012). We plotted *F*_ST_ values across the genome and identified the sites with the highest resulting *F*_ST_ values that are most characteristic for the respective species, and thus, might be involved in the adaptation to its habitat. At the sites with the highest *F*_ST_, we then calculated the allele frequency in each species and at each timepoint in angsd 0.925 (Korneliussen et al., 2014) after the method from Kim et al. (2011) to track the change in allele frequency differences over time. To represent sites most characteristic for the respective species, we selected either those sites with the top 10 highest *F*_ST_ values, the top 50 highest *F*_ST_ values, or all sites in the tail of the *F*_ST_ value distribution (using an empirical *p*‐value cutoff of 0.001). Additionally, we calculated the allele frequencies in each species and at each timepoint for the SNP (position 30197713 on scaffold 23) within the gene *edar* that has been found to be significantly associated with gill‐raker count (De‐Kayne et al., 2022), a trait that is relevant for the feeding ecology of each species. We further blasted the protein sequence of the gene *vgll3*, which is known to be relevant for age at maturity in *Salmo salar* (Barson et al., 2015), against the Alpine whitefish genome and found two equivalent best hits. For any SNPs in these two genes (likely paralogous copies of *vgll3*), we as well calculated the allele frequencies in each species and each timepoint to document the change in allele frequencies over time.

## 3 RESULTS

**FIGURE 1:**
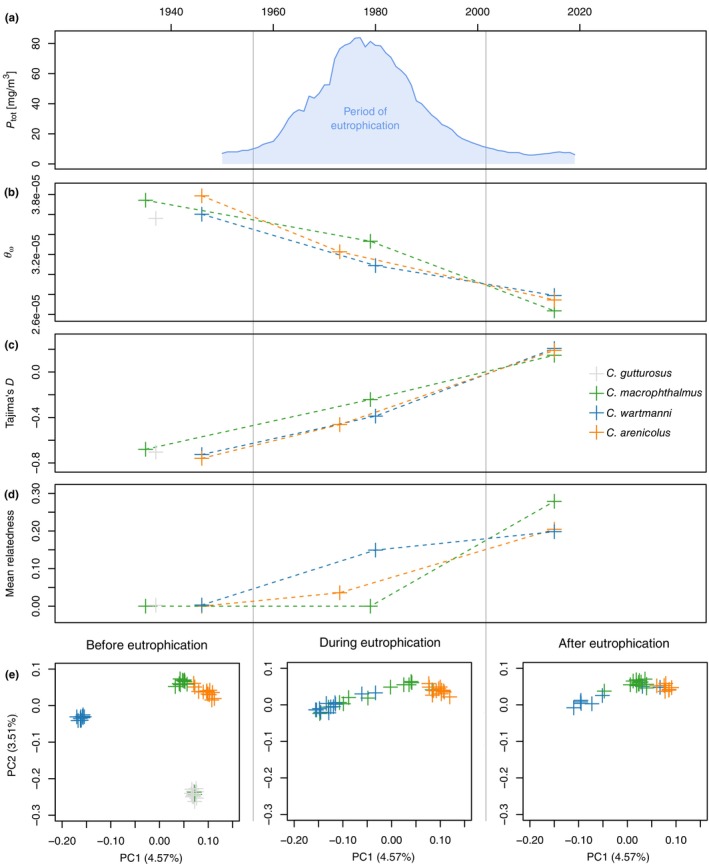
Total phosphate, nucleotide diversity, Tajima's *D* and relatedness over time. Total phosphate concentration over time (a), Watterson's theta (b) and Tajima's *D* (c), mean relatedness between individuals of each species and timepoint (d) and PCA based on genotype likelihoods separately plotted for each timepoint (e). (e) PCA showing the population structure of the Lake Constance whitefish radiation over time. These plots show the PCA from Figure S1, subsetted to the three sampling timepoints. Colours correspond to species (grey *C. gutturosus*, green *C. macrophthalmus*, blue *C. wartmanni* and orange *C. arenicolus*), see legend in panel (c).

We used natural history collections to sequence population genomic time series data, including an entire adaptive radiation and capturing a period of transient but severe ecological disturbance with the aim of documenting the influence of ecological disturbance on the genomic diversity of each single species, but also on the entire adaptive radiation (Figure 1).

### 3.1 Population structure

We performed a PCA based on genotype likelihoods of 128,164 SNPs to visualize population structure of the Lake Constance whitefish radiation over time, respectively, over the period of anthropogenic eutrophication (Figure 1e). Apart from the extinction of *C. gutturosus*, the three extant species qualitatively cluster closer together post‐eutrophication compared to pre‐eutrophication, suggesting that the species are today less differentiated than before the onset of eutrophication. This might be the consequence of interspecific hybridization during the eutrophication period as demonstrated in previous work (Frei, De‐Kayne, et al., 2022; Frei, Reichlin, et al., 2022; Vonlanthen et al., 2012), also consistent with several potential early generation hybrids in the during‐eutrophication sampling time‐point.

### 3.2 Nucleotide diversity

Irrespective of species, nucleotide diversity (measured as Watterson's theta) declined over time (Figure 1b). In each species, nucleotide diversity was highest before the onset of anthropogenic eutrophication, and it was lowest post‐eutrophication, while the populations sampled during peak eutrophication indicated values of nucleotide diversity between the pre‐ and post‐eutrophication population of the species. In total, *C. wartmanni* lost ~23%, *C. arenicolus* lost ~28% and *C. macrophthalmus* lost ~30% of their original nucleotide diversity from before the onset of the eutrophication period.

### 3.3 Tajima's *D*

The genome‐wide average of Tajima's *D* of each species was negative before and during the period of anthropogenic eutrophication (Figure 1c), indicative of population expansion after a recent bottleneck. This might reflect the recent colonization and evolution of the radiation within Lake Constance, since the last glacial maximum 10,000–15,000 years ago. However, in each species, Tajima's *D* was positive (*D* > 0; Figure 1c) after the period of anthropogenic eutrophication ended, potentially indicating a sudden population contraction associated with the altered environmental conditions.

### 3.4 Mean relatedness

For each extant species of Lake Constance whitefish, we calculated the pairwise relatedness between all individual before, during and after the period of anthropogenic eutrophication (Figure S2). Consistent with a decrease in population size, mean relatedness increased in all species over the period of eutrophication. All the species showed mean relatedness values below 0.01 before the start of the eutrophication period, while mean relatedness ranged between ~0.2 and ~0.28 in the post eutrophication population of the three extant species (Figure 1d).

### 3.5 Frequency shifts over time

**FIGURE 2:**
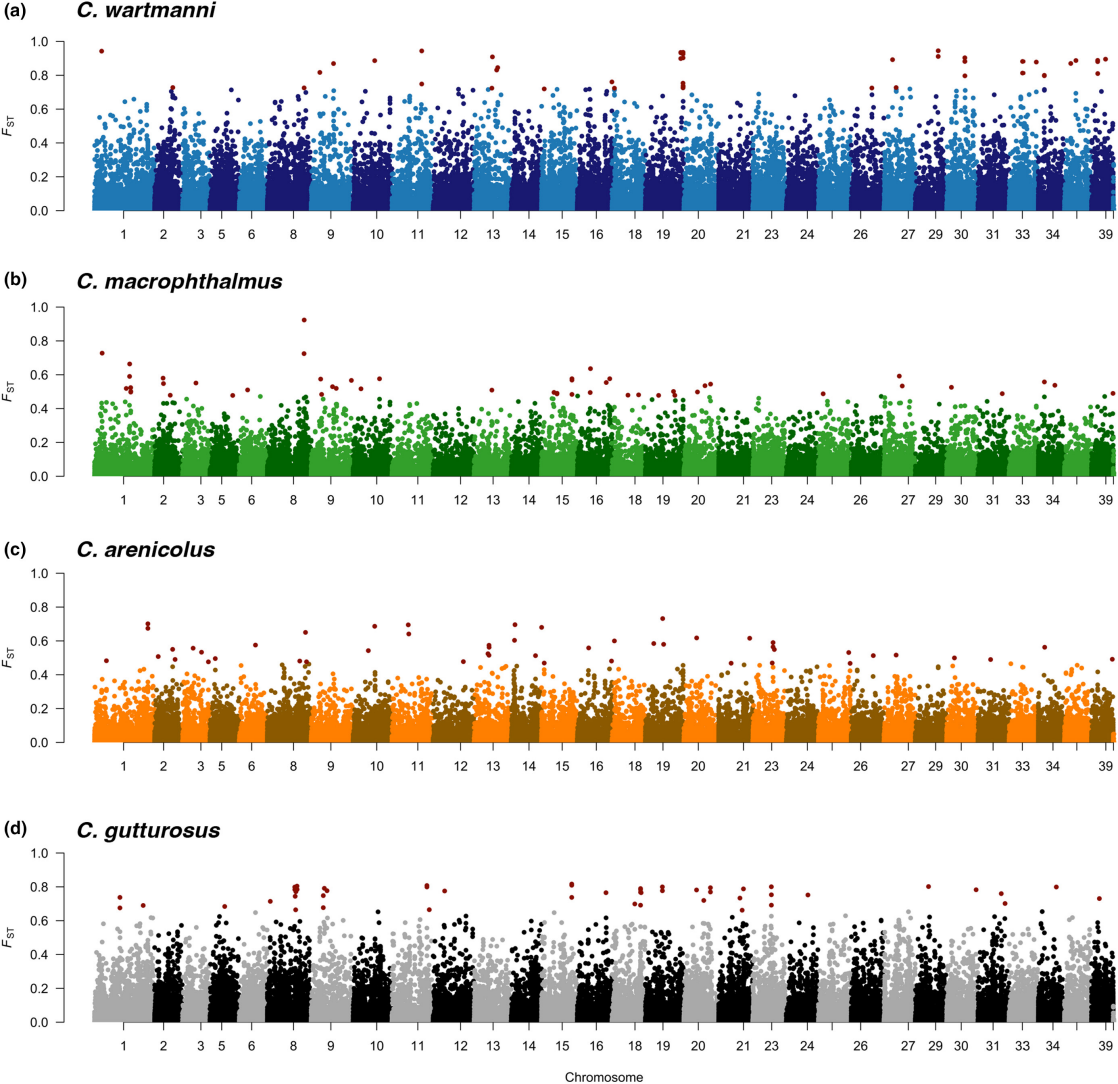
Genomic differentiation between cooccurring Alpine whitefish species before eutrophication of Lake Constance. (a) Genomic differentiation between *C. wartmanni* and all other species of the radiation. Differentiation (*F*_ST_ value) at the time point before eutrophication is plotted for each site across chromosomes along the genome. The 50 most characteristic sites are highlighted in red. (b) Genomic differentiation between *C. macrophthalmus* and all other species of the radiation. (c) Genomic differentiation between *C. arenicolus* and all other species of the radiation. (d) Genomic differentiation between the extinct *C. gutturosus* and all other species of the radiation.

**FIGURE 3:**
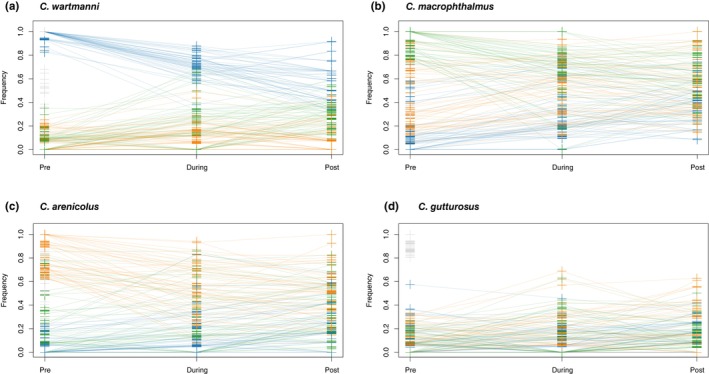
Allele frequency trajectories at most characteristic sites over time. (a) The trajectories of the 50 most characteristic sites of *C. wartmanni* (top 50 highest *F*_ST_ values when comparing *C. wartmanni* with all other species of the radiation at the time point before eutrophication). The allele frequencies of each position at the different sampling time‐points are connected with a line. The colour corresponds to species (blue *C. wartmanni*, green *C. macrophthalmus*, orange *C. arenicolus*). (b) The trajectories of the 50 most characteristic sites of *C. macrophthalmus*. (c) The trajectories of the 50 most characteristic sites of *C. arenicolus*. (d) The trajectories of the 50 most characteristic sites of the extinct *C. gutturosus*.

We identified the most characteristic alleles of each species by calculating the *F*_ST_ between each species and all other species pooled into one population at all SNPs along the genome (of only the pre‐eutrophication populations). For each pairwise comparison the 50 positions with the highest *F*_ST_ values were largely spread across different chromosomes (Figure 2). In line with previous work that identified few fixed differences between sympatric species of the Alpine whitefish radiation (De‐Kayne et al., 2022), we did not detect any fixed differences (*F*_ST_ >0.95). In all three extant species, we observed almost identical patterns of frequency trajectories at all of the 50 most characteristic sites: Allele frequency differences between species were homogenized over the period of eutrophication, because the frequency of the predominant allele in the focal species decreased, while its frequency in all other species increased over time (Figure 3). We observe qualitatively similar patterns when the top 10 most differentiated sites (Figure S3) or 356 sites in the tail of the distribution (Figure S4) were selected as most characteristic sites.

**FIGURE 4:**
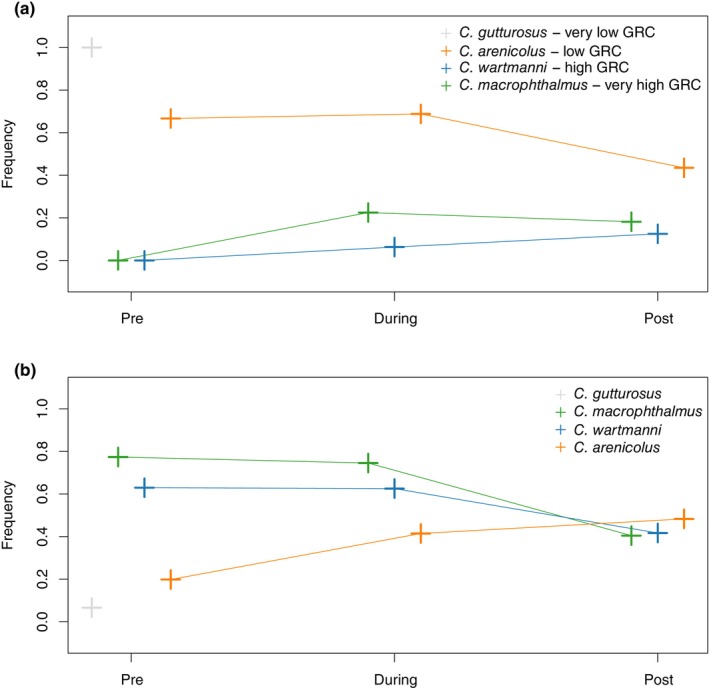
Allele frequency trajectory for two potentially functionally important loci. (a) Allele frequencies of the SNP found to be significantly associated with gill‐raker count by De‐Kayne et al. (2022). The legend shows the symbol and colour of each species, as well as the gill‐raker count of each species (very low, low, high, very high) according to De‐Kayne et al. (2022) and Vonlanthen et al. (2012). (b) Allele frequencies of the one SNP within the two vgll3 paralogs in the whitefish genome, where at least one of the pre‐eutrophication populations has a minor allele frequency above 0.15 and hence might reflect relevant genetic variation. *vgll3* has been found to control age at maturity in Atlantic salmon (Barson et al., 2015).

We also assessed the allele frequency change in two genes which affect ecologically relevant phenotypes in Salmonids. At a locus in the *edar* gene involved in determining the gill‐raker count of whitefish species from De‐Kayne et al. (2022), the pre‐eutrophication samples of the species with a low‐gill‐raker count indicated very high frequencies of the non‐reference allele (*C. gutturosus* 0.99 and *C. arenicolus* 0.66) while the species with a higher gill‐raker count (both *C. wartmanni* and *C. macrophthalmus*) were fixed for the reference allele (Figure 4a). After the eutrophication period ended, the differences between the surviving species became smaller (*C. macrophthalmus* increased from 0 to 0.18, *C. wartmanni* increased from 0.12, and *C. arenicolus* decreased from 0.66 to 0.43). We detected six polymorphic loci within two *vgll3* paralogs, a gene that is known to be involved in the age at maturity in *S. salar* (Barson et al., 2015; Czorlich et al., 2018). For five of these six loci, the allele frequencies varied only little between species and were very low (minor allele frequency below 0.15 across all species and time points). However, one SNP indicated a pattern where frequencies were differentiated between the species before eutrophication, but differentiation was completely lost after eutrophication (Figure 4b).

## 4 DISCUSSION

Genetic diversity is a core component of the adaptive potential of a population. As a result, the maintenance of genetic diversity is fundamental for fast adaptive responses to rapid environmental change. Hence, genetic diversity is a key component of the extinction risk of species during environmental change (Jensen & Leigh, 2022) and used as a metric to monitor threatened populations (Hoban et al., 2022). Anthropogenic ecological disturbance has the potential to decrease genetic diversity in natural populations (Sánchez‐Barreiro et al., 2021; Themudo et al., 2020). Here, we generated population level whole‐genome resequencing data of all extant species of an adaptive radiation before, during and after a severe but transient period of anthropogenic eutrophication. We tracked genomic diversity through time and found that genomic diversity was reduced after the period of eutrophication. Based on our results, we discuss the implications of reduced genetic diversity for the adaptive potential and extinction risk, as well as the relevance of such data for management and conservation.

### 4.1 Population‐decline during anthropogenic eutrophication

We observed substantial declines of genetic diversity in each species of the radiation over time, presumably in response to anthropogenic ecological disturbance of the ecosystem. In parallel, we observed a shift in Tajima's *D* from negative to positive in all species. Such change in genome‐wide Tajima's *D* suggests that rare alleles were lost over time, a pattern expected in declining populations. Furthermore, also in line with a decreasing population size, relatedness within each species increased over the period of eutrophication. Our estimates of relatedness likely are biased due to the limited sample size of the different species at specific timepoints (*N* = 5–13). Small sample sizes like in this study are expected to underestimate relatedness and bias might be more or less proportional to sample size (Wang, 2017). Hence, while absolute values are biased, trends through time might still provide relevant insights.

Taken together these results suggest that the period of anthropogenic eutrophication resulted in demographic decline of all three species, probably due to habitat loss and shifts in the available prey community (see Vonlanthen et al., 2012). Such a reduction in population sizes might potentially have strengthened genetic drift, and eventually resulted in the loss of substantial amounts of genomic variation in a very short period of time (~30 years or ~6 whitefish generations (Nussle et al., 2009)). It has been theoretically predicted that when genomic diversity declines linearly, population sizes might still be relatively large (Jensen & Leigh, 2022). When population sizes are declining fast, many rare alleles are lost rapidly, leading to strong initial loss of genomic diversity. After this initial loss of rare alleles, all remaining alleles become common and thus, it becomes harder to lose further variation via drift, slowing down the decline of genomic diversity. Hence, when populations sizes become small, the loss of genomic diversity is L‐shaped or exponential (Jensen & Leigh, 2022). We here observed rather linear declines of genomic diversity in all three studied species over time, in addition to a shift in genome‐wide Tajima's *D* consistent with a loss of rare alleles. This suggests that despite the population sizes declined during the period of eutrophication, each species might have still retained a relatively large effective population size. Estimates of pairwise nucleotide diversity *θ*_ω_ of 3.8 × 10^−5^ before eutrophication imply an effective population size *N*_e_ of about 947 (*N*_e_ = *θ*_ω_/4*μ*; assuming a mutation rate of 10^−8^ following Rougeux et al., 2017), with N_e_ dropping slightly to 833 (*θ*_ω_ of 3.3 × 10^−5^) during and to 686 (*θ*_ω_ of 2.7 × 10^−5^) after eutrophication (estimates for *C. arenicolus*, see Table S2 for qualitatively similar values of the other species).

### 4.2 Evolutionary rescue enabled by hybridization

Previous work showed that all the Lake Constance whitefish species extensively hybridized during the period of anthropogenic eutrophication, while there was little to no selection against genomic variation derived from hybridization (Frei, De‐Kayne, et al., 2022). Even though hybridization might not have been able to counteract the loss of genomic diversity caused by the population declines during eutrophication, it could have helped to maintain diversity in each species and to overcome the negative effects of reduced genomic variation. Through such a scenario of evolutionary rescue, interspecific hybridization might become adaptive when populations rapidly decline (Stelkens et al., 2014; Vedder et al., 2022). This suggests that when environments rapidly change, the risk of extinction of species that have evolved complete reproductive isolation against all other sympatric species might be higher than that of species that are still able to hybridize with one or several other species. Hence, the evolution of complete reproductive isolation of a species can become obstructive for the survival of the species under rapid environmental change.

### 4.3 Frequency differences homogenized in all species

We identified the 50 historically most characteristic SNPs of each species (50 highest *F*_ST_ values between the pre‐eutrophication population of each species and the pre‐eutrophication populations of all other species pooled together). These positions have been chosen to reflect some of the adaptation to each species' habitat that have evolved over the course of evolution (da Silva Riberio et al., 2022). When we compared the allele frequencies at these sites before, during and after eutrophication, we find that the frequency differences become homogenized over the period of eutrophication. Often, the frequency of the predominant allele in the focal species decreased, while its frequency in all other species increased over time, suggesting that the homogenization of allele frequency differences happened in all species. Two ecologically relevant genes, the *edar* locus associated with gill‐raker count from De‐Kayne et al. (2022) and paralogs of *vgll3* which is involved in the determination of age at maturity in Atlantic salmon (Barson et al., 2015; Czorlich et al., 2018), indicated the same pattern of homogenized interspecific allele frequency differences over the period of anthropogenic eutrophication. The loss of frequency differences at ecologically relevant loci is in line with phenotypic data showing reduced ranges of gill‐raker numbers after eutrophication (Vonlanthen et al., 2012). Furthermore, the homogenization of allele frequency differences at ecologically relevant alleles is consistent with extensive hybridization during the period of anthropogenic eutrophication, presumably in combination with reduced divergent selection between species. Hence, parts of the original species differentiation that might have evolved in response to adaptation to the selective pressure in the habitats of each species have potentially been lost as consequence of anthropogenic eutrophication. We here qualitatively describe patterns of allele frequency change, restraining from any qualitative evaluations due to our moderate sample sizes (*N* = 5–13) and low‐sequencing coverage (average coverage around 3–4×; see Table S1 for mean coverage per individual and Table S2 for average coverage for each species and timepoint). A simulation study exploring the correlation between estimated and true allele frequencies across different sample sizes and sequencing coverage, approximated a correlation of *r* ^2^ = 0.866 for a sample size of 10 and coverage of 4×, parameters close to our study (Lou et al., 2021). Hence, we acknowledge that our sampling design prevents exact estimates of allele frequencies, but may permit to observe general trends consistence across many loci widely distributed across the genome.

### 4.4 Whole‐genome resequencing conflicts with microsatellite results

While Vonlanthen et al. (2012) describe an increase in allelic richness of nine microsatellite markers over the period of eutrophication, using samples from the same historical scale collection as we used for this study, we here report substantial losses of genetic diversity based on whole‐genome resequencing data. This difference might be explained by the different data types. Microsatellites are multiallelic markers with a high‐mutation rate, resulting in fixed differences and private alleles between species or populations in a relatively short time span. However, in evolutionary young adaptive radiations, such as the Alpine whitefish radiation, species differentiation is mainly based on frequency shifts of thousands of SNPs. As a result of this difference between microsatellite and SNP markers, demographic‐ (such as population declines) or evolutionary processes (such as hybridization) can have different outcomes on diversity estimates based on the two different marker types. Hybridization between two species whose differentiation is based on moderate frequency shifts at many SNPs might have little impact on their nucleotide diversity, while allelic richness at microsatellites is greatly increased because new alleles were brought into the species that were beforehand private to the other species. If such a hybridization event is taking place during a period of weak population decline, nucleotide diversity at all SNPs might decrease (from demographic decline while hybridization has little effect), whilst allelic richness at microsatellite loci is increasing (because hybridization is bringing in more new alleles than are lost due to drift during population decline). The contrasting results between microsatellite loci, still often used in the context of conservation and management of natural populations and whole‐genome resequencing data, highlight the importance of marker choice to draw valid and robust conclusions for the question of interest. Considering the unprecedented contemporary rates of habitat loss and species extinctions, mitigating the consequences of genome‐wide losses of genetic variation is central for overcoming the current biodiversity crisis (Kardos et al., 2021) and only possible by making use of genomic data for conservation purposes (Supple & Shapiro, 2018).

### 4.5 The relevance of genomic long‐term data for biodiversity conservation

Natural populations need genomic diversity to maintain the evolutionary potential enabling a rapid evolutionary response to changing environments (Hoffmann et al., 2017). The erosion of a substantial amount of genomic variation within few generations in combination with the loss of potentially adaptive genomic differentiation, both evolved over thousands of years, demonstrates the sensitivity of evolutionary young adaptive radiations to environmental disturbance. Genetic erosion might have reduced the potential for resilience to future environmental change through a reduced evolutionary potential, increasing the extinction risk of each species. Therefore, characterizing the genomic change of natural populations across periods of ecological disturbance is fundamental to enhance species conservation and advance our understanding of biodiversity dynamics.

Understanding the genomic consequences of environmental change and its temporal dynamics heavily relies on a suitable baseline from before the onset of environmental disturbance (Jensen & Leigh, 2022). Increasing levels of ecosystem degradation on a global scale can result in lowered subjective threshold for acceptable environmental conditions. Without any historical records about the original condition of a given environment, new generations might consider the situation in which they have been raised as the appropriate baseline level (Soga & Gaston, 2018), a phenomenon termed the ‘shifting baselines syndrome’ (Pauly, 1995). Natural history collections, such as the one used here, can provide suitable baselines unaffected by anthropogenic influences and are therefore fundamental to counteract the shifting baselines syndrome. However, although it contributes disproportionally to biodiversity conservation and policy, the investment in generating long‐term data is declining (Hughes et al., 2017). Thus, the generation of genomic data from historical samples representing an appropriate baseline can fundamentally improve our understanding of the evolutionary response of natural populations to anthropogenic disturbance and thereby advance the establishment of targeted and efficient conservation measures.

## CONFLICT OF INTEREST STATEMENT

The authors declare no conflicts of interest.

## Supporting information


Data S1

## Data Availability

The raw sequencing files are accessible on ENA SRA (PRJEB57733). Additional supporting data are deposited on the eawag research data institutional collection (10.25678/0009D5). Scripts used for data analysis are on GitHub (https://github.com/freidavid/lake_constance_time_series_data).
