# Supplementary material for: Ecological disturbance reduces genomic diversity across an Alpine whitefish adaptive radiation
Source: Evol Appl. 2023 Nov 23;17(2):e13617. doi: 10.1111/eva.13617 (PMC10853656; doi:10.1111/eva.13617)
Supplement: Supplementary file 1 — Data S1 [file EVA-17-e13617-s001.docx]

Supplementary Files

**Supplementary Figure S1: Principal component analysis including all samples and timpepoints.** The same PCA is plotted in Figure 1E, but pre-, during and post-eutrophication samples are split into different panels.

**Figure S2: Increased relatedness after the eutrophication period. A)** Distribution of pairwise relatedness between the extinct *C. gutturosus* individuals sequenced shown as violin plot. **B)** Distribution of pairwise relatedness between *C. arenicolus* individuals from before, during and after the anthropogenic eutrophication period shown as violin plots. **C)** Distribution of pairwise relatedness between *C. macrophthalmus* individuals from before, during and after the anthropogenic eutrophication period shown as violin plots. **D)** Distribution of pairwise relatedness between *C. wartmanni* individuals from before, during and after the anthropogenic eutrophication period shown as violin plots. Grey dots show individual data points. **
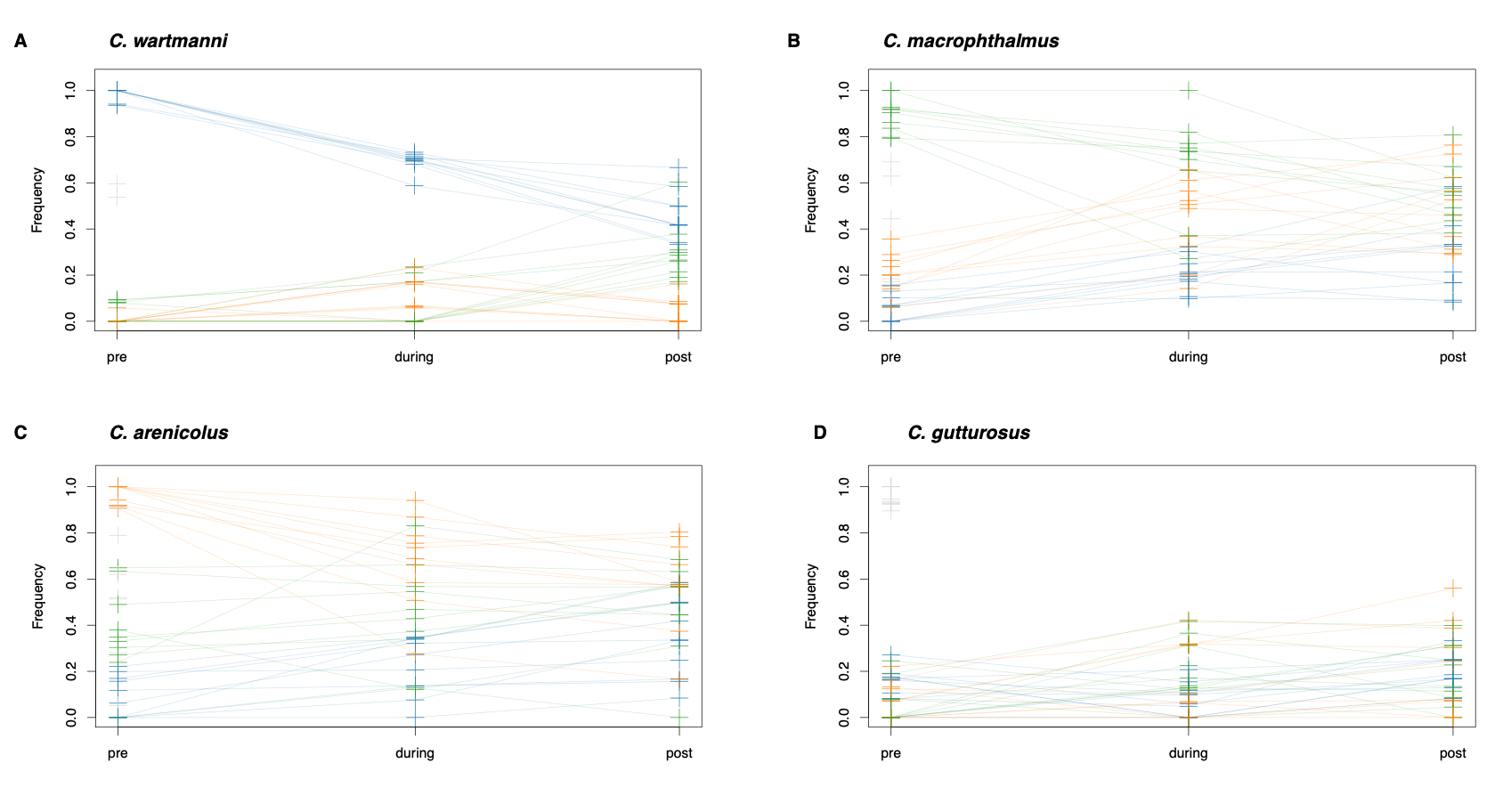
**

**Figure S3: Allele frequency trajectories at most characteristic sites over time. A)** The trajectories of the ten most characteristic sites of *C. wartmanni* (top ten highest *F*_ST_ values when comparing *C. wartmanni* with all other species of the radiation at the time point before eutrophication). The allele frequencies of each position at the different sampling time-points are connected with a dashed line. The color corresponds to species (blue *C. wartmanni*, green *C. macrophthalmus*, orange *C. arenicolus*). **B)** The trajectories of the ten most characteristic sites of *C. macrophthalmus.* **C)** The trajectories of the ten most characteristic sites of *C. arenicolus.* **D)** The trajectories of the ten most characteristic sites of the extinct *C. gutturosus.*

**
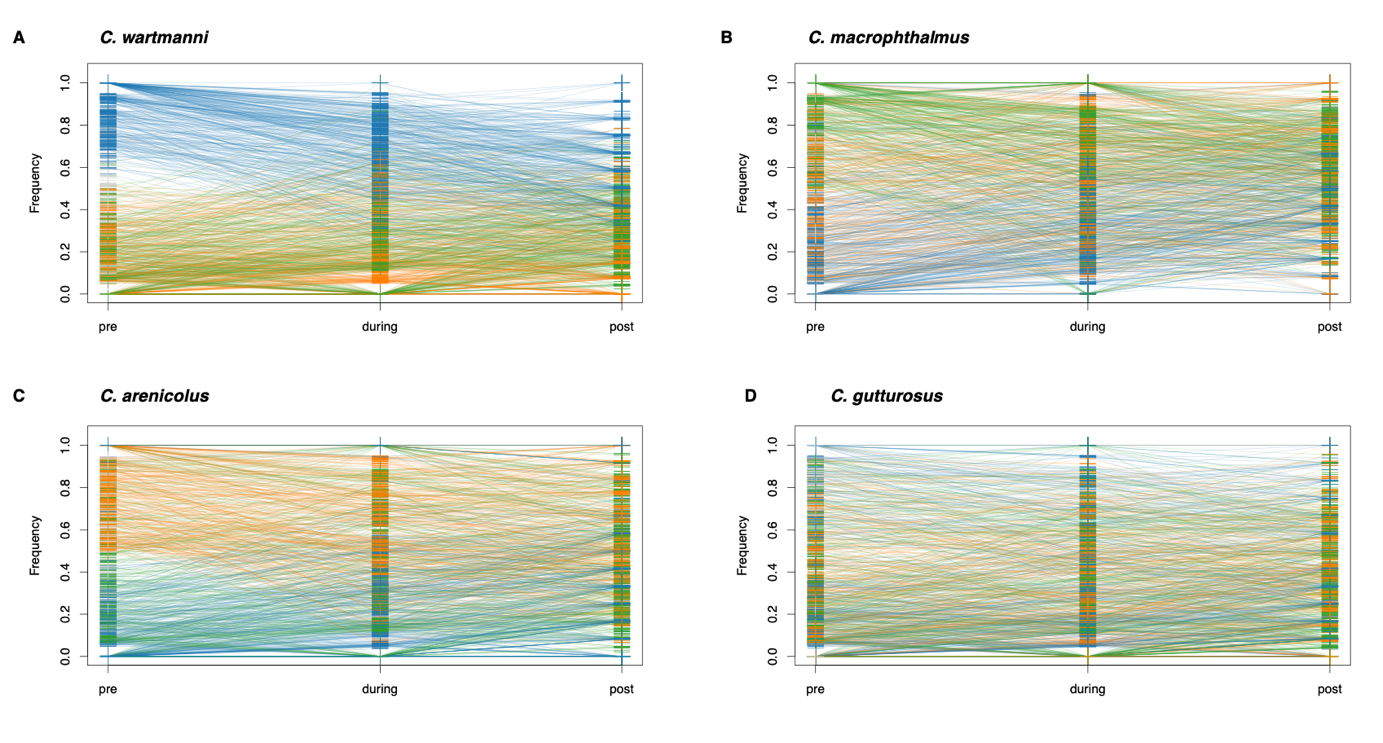
**

**Figure S4: Allele frequency trajectories at most characteristic sites over time. A)** The trajectories of the most characteristic sites of *C. wartmanni* (356 sites in the tail of the distribution of *F*_ST_ values when comparing *C. wartmanni* with all other species of the radiation at the time point before eutrophication). The allele frequencies of each position at the different sampling time-points are connected with a dashed line. The color corresponds to species (blue *C. wartmanni*, green *C. macrophthalmus*, orange *C. arenicolus*). **B)** The trajectories of the most characteristic sites of *C. macrophthalmus.* **C)** The trajectories of the most characteristic sites of *C. arenicolus.* **D)** The trajectories of the most characteristic sites of the extinct *C. gutturosus.*

**Supplementary Table S1: Overview over all 112 whole-genome sequenced samples.** The table lists species assignment of the sample when it was collected (“Species”), year of collection, sampling timepoint in relation to the eutrophication period (“Time”), species assignment based on genomic data (“Species Genomic”) and ENA sample accessions as well as the mean coverage (“Coverage”) for the respective sample. Twelve samples for which “Species” and “Species Genomic” columns do not match have been excluded from analyses.

| **Species** | **Year** | **Time** | **Species Genomic** | **Lab ID** | **ENA accession** | **Coverage** |
| --- | --- | --- | --- | --- | --- | --- |
| *C. wartmanni* | 2015 | post | *C. wartmanni* | 121 | ERS6670439 | 59.4 |
| *C. wartmanni* | 2015 | post | *C. wartmanni* | 122 | ERS6670440 | 47.5 |
| *C. wartmanni* | 2015 | post | *C. wartmanni* | 123 | ERS6670441 | 58.9 |
| *C. arenicolus* | 2015 | post | *C. arenicolus* | 126 | ERS6670448 | 61.7 |
| *C. arenicolus* | 2015 | post | *C. arenicolus* | 127 | ERS6670449 | 57.9 |
| *C. arenicolus* | 2015 | post | *C. arenicolus* | 128 | ERS6670450 | 46.4 |
| *C. wartmanni* | 2015 | post | *C. wartmanni* | 131 | ERS6670442 | 60.2 |
| *C. macrophthalmus* | 2015 | post | *C. macrophthalmus* | 132 | ERS6670445 | 40.5 |
| *C. macrophthalmus* | 2019 | post | *C. macrophthalmus* | 212608 | ERS12047271 | 8.2 |
| *C. macrophthalmus* | 2019 | post | *C. macrophthalmus* | 212623 | ERS12047279 | 8.0 |
| *C. arenicolus* | 2019 | post | *C. arenicolus* | 212631 | ERS12047284 | 9.4 |
| *C. arenicolus* | 2019 | post | *C. arenicolus* | 212633 | ERS12047285 | 10.3 |
| *C. arenicolus* | 2019 | post | *C. arenicolus* | 212634 | ERS12047286 | 7.2 |
| *C. macrophthalmus* | 2019 | post | *C. macrophthalmus* | 212663 | ERS12047313 | 4.3 |
| *C. macrophthalmus* | 2019 | post | *C. macrophthalmus* | 212666 | ERS12047316 | 18.6 |
| *C. macrophthalmus* | 2019 | post | *C. macrophthalmus* | 212671 | ERS12047319 | 21.5 |
| *C. macrophthalmus* | 2019 | post | *C. macrophthalmus* | 212672 | ERS12047320 | 6.1 |
| *C. macrophthalmus* | 2019 | post | *C. macrophthalmus* | 212678 | ERS12047326 | 9.5 |
| *C. macrophthalmus* | 2019 | post | *C. macrophthalmus* | 212694 | ERS12047341 | 6.4 |
| *C. macrophthalmus* | 2019 | post | *C. macrophthalmus* | 212695 | ERS12047342 | 5.3 |
| *C. macrophthalmus* | 2019 | post | *C. macrophthalmus* | 212696 | ERS12047343 | 6.7 |
| *C. arenicolus* | 2019 | post | *C. arenicolus* | 212701 | ERS12047348 | 6.7 |
| *C. wartmanni* | 2019 | post | *C. macrophthalmus* | 212702 | this study | 17.2 |
| *C. wartmanni* | 2019 | post | *C. arenicolus* | 212703 | this study | 22.6 |
| *C. wartmanni* | 2019 | post | *C. wartmanni* | 212704 | this study | 19.6 |
| *C. wartmanni* | 2019 | post | *C. wartmanni* | 212705 | this study | 18.7 |
| *C. macrophthalmus* | 2019 | post | *C. macrophthalmus* | 212718 | ERS12047354 | 6.4 |
| *C. macrophthalmus* | 2019 | post | *C. macrophthalmus* | 212725 | ERS12047361 | 8.5 |
| *C. macrophthalmus* | 2019 | post | *C. wartmanni* | 212727 | ERS12047363 | 10.5 |
| *C. gutturosus* | 1948 | pre | *C. gutturosus* | S01_0003_gutturosus_1948 | this study | 2.5 |
| *C. gutturosus* | 1948 | pre | *C. gutturosus* | S01_0006_gutturosus_1948 | this study | 2.4 |
| *C. gutturosus* | 1948 | pre | *C. gutturosus* | S01_0020_gutturosus_1948 | this study | 2.2 |
| *C. gutturosus* | 1948 | pre | *C. gutturosus* | S01_0025_gutturosus_1948 | this study | 2.3 |
| *C. gutturosus* | 1948 | pre | *C. gutturosus* | S01_0031_gutturosus_1948 | this study | 3.2 |
| *C. gutturosus* | 1948 | pre | *C. gutturosus* | S01_0039_gutturosus_1948 | this study | 2.6 |
| *C. arenicolus* | 1946 | pre | *C. arenicolus* | S11_0001_arenicolus_1946 | this study | 2.6 |
| *C. arenicolus* | 1946 | pre | *C. arenicolus* | S11_0002_arenicolus_1946 | this study | 2.9 |
| *C. arenicolus* | 1946 | pre | *C. arenicolus* | S11_0003_arenicolus_1946 | this study | 2.7 |
| *C. arenicolus* | 1946 | pre | *C. arenicolus* | S11_0004_arenicolus_1946 | this study | 2.6 |
| *C. arenicolus* | 1946 | pre | *C. arenicolus* | S11_0005_arenicolus_1946 | this study | 1.8 |
| *C. arenicolus* | 1946 | pre | *C. arenicolus* | S11_0009_arenicolus_1946 | this study | 2.3 |
| *C. arenicolus* | 1946 | pre | *C. arenicolus* | S11_0010_arenicolus_1946 | this study | 1.9 |
| *C. arenicolus* | 1946 | pre | *C. arenicolus* | S11_0014_arenicolus_1946 | this study | 2.4 |
| *C. arenicolus* | 1946 | pre | *C. arenicolus* | S11_0015_arenicolus_1946 | this study | 2.2 |
| *C. arenicolus* | 1946 | pre | *C. arenicolus* | S11_0016_arenicolus_1946 | this study | 2.1 |
| *C. arenicolus* | 1946 | pre | *C. arenicolus* | S11_0017_arenicolus_1946 | this study | 2.3 |
| *C. arenicolus* | 1946 | pre | *C. arenicolus* | S11_0019_arenicolus_1946 | this study | 2.4 |
| *C. wartmanni* | 1946 | pre | *C. wartmanni* | S12_0400_wartmanni_1946 | this study | 2.3 |
| *C. wartmanni* | 1946 | pre | *C. wartmanni* | S12_0402_wartmanni_1946 | this study | 3.4 |
| *C. wartmanni* | 1946 | pre | *C. wartmanni* | S12_0403_wartmanni_1946 | this study | 3.2 |
| *C. wartmanni* | 1946 | pre | *C. wartmanni* | S12_0404_wartmanni_1946 | this study | 3.0 |
| *C. wartmanni* | 1946 | pre | *C. wartmanni* | S12_0406_wartmanni_1946 | this study | 2.8 |
| *C. wartmanni* | 1946 | pre | *C. wartmanni* | S12_0409_wartmanni_1946 | this study | 2.6 |
| *C. wartmanni* | 1946 | pre | *C. wartmanni* | S12_0410_wartmanni_1946 | this study | 2.4 |
| *C. wartmanni* | 1946 | pre | *C. wartmanni* | S12_0411_wartmanni_1946 | this study | 2.9 |
| *C. wartmanni* | 1946 | pre | *C. wartmanni* | S12_0414_wartmanni_1946 | this study | 2.4 |
| *C. wartmanni* | 1946 | pre | *C. wartmanni* | S12_0415_wartmanni_1946 | this study | 2.9 |
| *C. wartmanni* | 1946 | pre | *C. wartmanni* | S12_0417_wartmanni_1946 | this study | 3.4 |
| *C. wartmanni* | 1946 | pre | *C. wartmanni* | S12_0419_wartmanni_1946 | this study | 2.8 |
| *C. arenicolus* | 1973 | during | *C. arenicolus* | S13_0001_arenicolus_1973 | this study | 5.5 |
| *C. arenicolus* | 1973 | during | *C. arenicolus* | S13_0002_arenicolus_1973 | this study | 3.4 |
| *C. arenicolus* | 1973 | during | *C. arenicolus* | S13_0005_arenicolus_1973 | this study | 3.4 |
| *C. arenicolus* | 1973 | during | *C. arenicolus* | S13_0011_arenicolus_1973 | this study | 1.4 |
| *C. arenicolus* | 1973 | during | *C. arenicolus* | S13_0012_arenicolus_1973 | this study | 0.7 |
| *C. arenicolus* | 1973 | during | *C. arenicolus* | S13_0013_arenicolus_1973 | this study | 4.6 |
| *C. arenicolus* | 1973 | during | *C. arenicolus* | S13_0015_arenicolus_1973 | this study | 2.8 |
| *C. arenicolus* | 1973 | during | *C. arenicolus* | S13_0016_arenicolus_1973 | this study | 2.5 |
| *C. arenicolus* | 1973 | during | *C. arenicolus* | S13_0017_arenicolus_1973 | this study | 3.5 |
| *C. arenicolus* | 1973 | during | *C. arenicolus* | S13_0018_arenicolus_1973 | this study | 4.2 |
| *C. arenicolus* | 1973 | during | *C. arenicolus* | S13_0019_arenicolus_1973 | this study | 5.4 |
| *C. arenicolus* | 1973 | during | *C. arenicolus* | S13_0020_arenicolus_1973 | this study | 4.7 |
| *C. wartmanni* | 1980 | during | *C. wartmanni* | S16_0006_wartmanni_1980 | this study | 4.6 |
| *C. wartmanni* | 1980 | during | *C. wartmanni* | S16_0010_wartmanni_1980 | this study | 5.0 |
| *C. wartmanni* | 1980 | during | *C. wartmanni* | S16_0011_wartmanni_1980 | this study | 3.9 |
| *C. wartmanni* | 1980 | during | *C. wartmanni* | S16_0012_wartmanni_1980 | this study | 5.3 |
| *C. wartmanni* | 1980 | during | *C. wartmanni* | S16_0013_wartmanni_1980 | this study | 4.7 |
| *C. wartmanni* | 1980 | during | *C. wartmanni* | S16_0014_wartmanni_1980 | this study | 8.6 |
| *C. wartmanni* | 1980 | during | *C. wartmanni* | S16_0015_wartmanni_1980 | this study | 4.5 |
| *C. wartmanni* | 1980 | during | *C. wartmanni* | S16_0016_wartmanni_1980 | this study | 5.2 |
| *C. wartmanni* | 1980 | during | *C. wartmanni* | S16_0017_wartmanni_1980 | this study | 5.0 |
| *C. wartmanni* | 1980 | during | *C. wartmanni* | S16_0018_wartmanni_1980 | this study | 6.0 |
| *C. wartmanni* | 1980 | during | *C. wartmanni* | S16_0020_wartmanni_1980 | this study | 5.1 |
| *C. wartmanni* | 1980 | during | *C. wartmanni* | S16_0023_wartmanni_1980 | this study | 2.8 |
| *C. macrophthalmus* | 1935 | pre | *C. macrophthalmus* | S19_0026_macrophthalmus_1935 | this study | 3.0 |
| *C. macrophthalmus* | 1935 | pre | *C. macrophthalmus* | S19_0030_macrophthalmus_1935 | this study | 3.2 |
| *C. macrophthalmus* | 1935 | pre | *C. macrophthalmus* | S19_0032_macrophthalmus_1935 | this study | 3.2 |
| *C. macrophthalmus* | 1935 | pre | *C. macrophthalmus* | S19_0035_macrophthalmus_1935 | this study | 4.3 |
| *C. macrophthalmus* | 1935 | pre | *C. macrophthalmus* | S19_0036_macrophthalmus_1935 | this study | 2.7 |
| *C. macrophthalmus* | 1935 | pre | *C. macrophthalmus* | S19_0037_macrophthalmus_1935 | this study | 3.6 |
| *C. macrophthalmus* | 1935 | pre | *C. macrophthalmus* | S19_0038_macrophthalmus_1935 | this study | 3.4 |
| *C. macrophthalmus* | 1935 | pre | *C. macrophthalmus* | S19_0039_macrophthalmus_1935 | this study | 3.3 |
| *C. macrophthalmus* | 1935 | pre | *C. macrophthalmus* | S19_0040_macrophthalmus_1935 | this study | 2.9 |
| *C. macrophthalmus* | 1935 | pre | *C. gutturosus* | S19_0046_macrophthalmus_1935 | this study | 3.5 |
| *C. macrophthalmus* | 1935 | pre | *C. gutturosus* | S19_0050_macrophthalmus_1935 | this study | 3.3 |
| *C. macrophthalmus* | 1979 | during | *C. macrophthalmus* | S19_0098_macrophthalmus_1979 | this study | 2.8 |
| *C. macrophthalmus* | 1979 | during | *C. wartmanni* | S19_0102_macrophthalmus_1979 | this study | 3.5 |
| *C. macrophthalmus* | 1979 | during | *C. macrophthalmus* | S19_0103_macrophthalmus_1979 | this study | 3.6 |
| *C. macrophthalmus* | 1979 | during | *C. macrophthalmus* | S19_0104_macrophthalmus_1979 | this study | 3.6 |
| *C. macrophthalmus* | 1979 | during | *C. macrophthalmus* | S19_0107_macrophthalmus_1979 | this study | 3.7 |
| *C. macrophthalmus* | 1979 | during | *C. macrophthalmus* | S19_0108_macrophthalmus_1979 | this study | 3.8 |
| *C. macrophthalmus* | 1979 | during | *C. arenicolus* | S19_0109_macrophthalmus_1979 | this study | 3.1 |
| *C. macrophthalmus* | 1979 | during | *C. wartmanni* | S19_0110_macrophthalmus_1979 | this study | 3.0 |
| *C. macrophthalmus* | 1979 | during | *C. wartmanni* | S19_0111_macrophthalmus_1979 | this study | 4.0 |
| *C. macrophthalmus* | 1979 | during | *C. wartmanni* | S19_0112_macrophthalmus_1979 | this study | 3.5 |
| *C. macrophthalmus* | 1979 | during | *C. wartmanni* | S19_0113_macrophthalmus_1979 | this study | 4.1 |
| *C. macrophthalmus* | 1979 | during | *C. wartmanni* | S19_0114_macrophthalmus_1979 | this study | 2.9 |
| *C. gutturosus* | 1937 | pre | *C. gutturosus* | S20_0051_gutturosus_1937 | this study | 3.1 |
| *C. gutturosus* | 1937 | pre | *C. gutturosus* | S20_0068_gutturosus_1937 | this study | 3.2 |
| *C. gutturosus* | 1937 | pre | *C. gutturosus* | S20_0069_gutturosus_1937 | this study | 2.5 |
| *C. gutturosus* | 1937 | pre | *C. gutturosus* | S20_0075_gutturosus_1937 | this study | 3.0 |
| *C. gutturosus* | 1937 | pre | *C. gutturosus* | S20_0079_gutturosus_1937 | this study | 3.6 |
| *C. gutturosus* | 1937 | pre | *C. gutturosus* | S20_0080_gutturosus_1937 | this study | 2.6 |

**Supplementary Table S2: Sample sizes and summary statistics for each species and timepoint.** For each species (“Species”) the year of sampling (“Year”), timepoint in relation to the eutrophication period (“Time”), and the sample size (“N”) as well average coverage across those samples (“coverage”) is given. Further the summary statistics as plotted in Figure 1 are listed Watterson’s theta ("theta”), Tajima’s D (“D”), and mean relatedness (“relatedness”) between individuals of each species and timepoint, as well as the inferred effective populations sizes (“Ne”) based on a mutation rate of
10^-8^ (following Rougeux, Bernatchez, & Gagnaire 2017).

| **Species** | **Year** | **Time** | **N (coverage)** | **theta** | **D** | **relatedness** | **Ne** |
| --- | --- | --- | --- | --- | --- | --- | --- |
| *C. arenicolus* | 1948 | pre | 12 (2.3) | 0.00003786 | -0.76 | 0.00002 | 947 |
| *C. arenicolus* | 1973 | during | 12 (3.5) | 0.00003332 | -0.46 | 0.03576 | 833 |
| *C. arenicolus* | 2015 | post | 7 (27.8) | 0.00002745 | 0.19 | 0.20462 | 686 |
| *C. macrophthalmus* | 1935 | pre | 9 (3.3) | 0.00003742 | -0.68 | 0.00013 | 936 |
| *C. macrophthalmus* | 1979 | during | 5 (3.5) | 0.00003332 | -0.24 | 0.00001 | 833 |
| *C. macrophthalmus* | 2019 | post | 13 (11.9) | 0.00002636 | 0.15 | 0.27886 | 659 |
| *C. wartmanni* | 1946 | pre | 12 (2.8) | 0.00003602 | -0.73 | 0.00307 | 901 |
| *C. wartmanni* | 1980 | during | 12 (4.5) | 0.00003332 | -0.39 | 0.14920 | 833 |
| *C. wartmanni* | 2015-2019 | post | 6 (39.3) | 0.00002790 | 0.21 | 0.19818 | 697 |
| *C. gutturosus* | 1937-1948 | pre | 12 (2.9) | 0.00003560 | -0.70 | 0.00242 | 890 |
